# Harnessing education and lifestyle change to support transitional health for returning citizens: a feasibility study protocol

**DOI:** 10.1186/s40814-023-01369-0

**Published:** 2023-08-09

**Authors:** Rodlescia S. Sneed, Leon El-Alamin, Marsha Thrower, Jessica Nadrowski, Kayla Habermehl

**Affiliations:** 1 Charles Stewart Mott Department of Public Health, Michigan State University, 200 East 1st Street, MI 48502 Flint, USA; 2 MADE Institute, 503 East Garland St, Flint, MI 48502 USA

**Keywords:** Formerly incarcerated, Returning citizens, Feasibility study, Chronic disease prevention, Physical activity, Nutrition

## Abstract

**Background:**

Exercise and healthy eating are known to reduce chronic disease risk; however, formerly incarcerated individuals (i.e., returning citizens) face significant social barriers when attempting to engage with existing community-based physical activity and nutrition programs. Given the high disease burden and unique educational needs of returning citizens, this proposed work fills an important gap in the support services currently offered to this population.

**Methods:**

This article describes a process for evaluating the feasibility and acceptability of a physical activity and nutrition intervention tailored to the needs of returning citizens via a partnership between an academic research organization and a community-based reentry program for returning citizens. Staff from the community-based reentry program will train four returning citizens as group fitness instructors and work with a nutritionist to design a 16-week nutrition education curriculum. Reentry program staff will enroll up to 15 formerly incarcerated adults (aged 18 +) in the exercise and nutrition program. Participants will take part in weekly nutrition classes led by the nutritionist and weekly group exercise classes led by a peer instructor. Research staff will evaluate program success in four domains: reach, preliminary effectiveness, implementation, and maintenance.

**Results:**

This feasibility study will allow us to design and test a program that can eventually be evaluated in a large, randomized trial. It also addresses the multitude of social determinants that impact the health of returning citizens and builds a framework for program sustainability. Individuals recruited as fitness trainers will acquire skills and certifications that may be used to sustain physical fitness activities within the reentry program.

**Conclusions:**

This feasibility study will test our ability to address social determinants that impact the health of returning citizens through a peer-led physical activity and nutrition programming. In the long term, study results may inform development and implementation of reentry programming and policy on a wider scale.

## Background

The number of individuals released from state and federal prison has increased substantially in recent years. In the year 2000, roughly 600,000 individuals were released [1]. By 2008, that number had grown to more than 730,000, representing an increase of more than 21% over an eight-year period [1]. In the USA, about 5 million formerly incarcerated adults (i.e., returning citizens) are under community supervision (e.g., probation or parole) at any given time [2].

Individuals who return to community settings after incarceration experience high rates of chronic disease and disability. Sixty-four percent of individuals ages 45 + in state and federal prisons report having a current medical problem [3]. Persons in state or federal prisons are 1.5 times more likely than persons in the general U.S. population to report ever having a chronic condition, and half of all persons in state or federal prisons report having a chronic condition such as asthma, heart disease, or diabetes [3]. Given that 95% of incarcerated individuals eventually return to community settings, this translates into a high burden of chronic disease upon community reentry [2]. After release, these returning citizens often come back to economically vulnerable communities where they seek support from local programs designed to help them successfully transition back to community life.

Currently, there is little attention to chronic disease prevention and self-management in community reentry programs, despite high rates of chronic disease among returning citizens. Most community reentry programming focuses on mental health, substance abuse, work readiness, and/or education [4]. The sparse programming that does exist related to physical health tends to focus on clinical care and management [5]. There is little, if any, attention paid to prevention and self-management of chronic physical health conditions through non-clinical approaches. Healthy eating and physical activity are known to reduce chronic disease risk, yet most reentry programs do not include physical activity and nutrition programming specifically designed with returning citizens in mind [6, 7].

Incorporating health-related programming into community reentry programs is crucial for addressing physical health among returning citizens. Returning citizens often face a great deal of social stigma and isolation when attempting to re-integrate into society [8]. Returning citizens frequently have limited social ties, as many intentionally separate from friends and family members who may have contributed to their incarceration. For those who have family, it may be difficult and stressful to re-integrate into the family unit, particularly if the circumstances resulting in incarceration occurred within the family or if the offense is deemed shameful by the family [9]. Thus, instead of relying on family support, many seek support from programs specifically designed for them [10]. Reentry programs, through their comprehensive programming, offer returning citizens a trusted system of support for navigating community life [10]. Many staff members of community reentry programs are returning citizens themselves; thus, they can understand the challenges and stigma faced by returning citizens and provide help in managing their health [10].

Existing physical activity and nutrition programs fail to address the needs of returning citizens. Returning citizens face numerous barriers that are often not addressed by physical activity and nutrition programs designed for the general population. For example, housing insecurity is an ongoing problem in this population [11]. Returning citizens are 10 times more likely to be homeless than the general population [11]. Thus, many must meet their nutritional needs while living in shelters or other transitional settings without kitchens or cooking equipment. Many returning citizens also lack the literacy skills needed to master traditional programming. Roughly 78% of incarcerated individuals have basic or below basic quantitative literacy, 50% have basic or below basic document literacy, and 56% have basic or below basic prose literacy [12]. Thus, most require programs that rely on live demonstrations and graphically appealing printed materials with limited text. Further, returning citizens experience high rates of poverty. One in five people returning from jail or prison earns less than $7,600 in the year following release, which is only about 1/7 of the U.S. median household income [13]. Given this, many returning citizens rely on public food assistance and food pantries to meet their nutritional needs. Traditional nutrition programs typically do not include strategies for using food stamps and food pantries to support healthy eating.

The purpose of the current study is to evaluate the feasibility and acceptability of a physical activity and nutrition program designed specifically to address the needs of returning citizens. This paper describes our planned process for an ongoing partnership between an academic research institution and a community-based reentry program [14]. Specifically, we will use a community-academic partnership approach to (1) evaluate the feasibility and acceptability of teaching returning citizens to be group fitness trainers who can lead physical activity workshops for their peers; (2) develop a nutrition curriculum tailored to the needs of returning citizens; and (3) use a single group exploratory study to evaluate the feasibility, acceptability, and preliminary effectiveness of a 16-week physical activity and nutrition program implemented among returning citizens who participate in activities run by the community-based reentry program.

## Materials and methods

### Inclusion/exclusion criteria

Individuals will be eligible for this study if they (1) are at least 18 years old, and (2) are current or former participants in the local reentry program implementing the project. Individuals will be excluded from participation if they (1) indicate that a doctor or nurse has told them not to engage in physical activity, (2) are unable or unwilling to provide informed consent.

### Informed consent

Individuals participating in the fitness instructor training program and/or the physical activity and nutrition program will complete an informed consent process. Study staff trained in human subjects protection will meet with potential participants to explain all aspects of the study, including the risks and benefits and obtain participants’ written informed consent. We will orally describe the material written in the informed consent document and answer any questions the participant may have. The participants will be reminded that they are not required to participate in the study and that their decision about whether to participate in the study will have no consequences. Participants who give their consent will sign a copy of the document and will be given a copy of the informed consent document. To accompany varying reading levels, consent forms will always be read aloud.

### Fitness instructor training program

Staff from a community-based reentry program will identify four returning citizens (two male, two female) to be trained as group fitness instructors who can provide instruction to their peers. These individuals will agree to participate in a 36-h training program (Table 1) that will include (1) cardiopulmonary resuscitation (CPR) training; (2) first aid training; (3) automated external defibrillator (AED) training; (4) group fitness certification; and (5) specialized training in the development of low-impact fitness classes. *CPR training* will equip instructors to respond to breathing or cardiac emergencies that could occur during fitness classes. *AED training* will equip instructors to use a defibrillator to assist individuals who experience cardiac arrest. First aid training will prepare trainees to recognize and care for a variety of first aid, breathing, and cardiac emergencies involving adults, children, and infants. The course features an interactive simulation learning experience where participants will respond to real-world emergencies. CPR, first aid, and AED training are routinely offered via the local Red Cross [15]. All three trainings satisfy Occupational Safety and Health Administration (OSHA) requirements. Upon completion of the trainings, students will receive a 2-year Red Cross certification.

**Table 1:** Fitness instructor training program overview

1) CPR training
• Checking the Scene and the Person
• Calling 911
• Opening the airway
• Checking for breathing
• Push hard, push fast
• Delivering rescue breaths
• Repeating CPR
2) First aid training
3) AED training
4) Group fitness certification
• Basic Fitness and Healthy Living Principles
• F.I.T. Principle and Basic Training Concepts
• Basic Cardiovascular and Neuromuscular Science Principles
• Basic Nutrition and Weight Management
• American College of Sports Medicine Guidelines, Exercise Safety & Injury Prevention
• Class Format, Cueing, Music and Choreography
• Basic Class Formats
5) Low-impact fitness training
• Warm-up
• Moderate intensity aerobics
• Resistance training (with cuff weights)
• Flexibility training
• Cool down

Trainees will participate in a *Group Fitness Certification* Program offered through the local YMCA [16]. This program will provide trainees with the tools necessary to teach a safe and effective fitness class in a group setting (Table 1). After completing the program, participants will complete the Group Fitness Instructor Certification Exam. Finally, trainees will participate in specialized training focused on the development of low-impact fitness classes [17]. This 12-h (1.5 day) training program focuses on dynamic cardiovascular exercise, strength training, balance, and flexibility. The training includes audiovisual material and slides. The instruction closely follows a written Instructor Manual that the new instructor keeps for reference. Day 2 of the training includes teach-back sessions in which the new instructor can demonstrate that they have learned the program protocols. In addition to leading the teach-back training, a certified group fitness coach also will assist the trainees in developing their own customized, 1-h class to be taught to their peers. Trainees will have both written and supplemental audiovisual materials to assist them during the training process.

### Nutrition curriculum development

We will work with a local nutritionist to develop a 16-week nutrition education curriculum to later be implemented among 15 returning citizens involved in reentry programming. Members of our team previously developed a 16-week nutrition program that was successfully utilized among more than 250 adults who participated in the Church Challenge, a physical activity and nutrition program conducted in Flint’s African-American faith community [18]. The Church Challenge nutrition program included instruction on high blood pressure prevention and control, eating a low sodium diet, food groups, serving sizes, meal and grocery planning, reading nutrition labels, and navigating the grocery store [18].

We will modify this program in two important ways. First, given that many justice-involved individuals have basic or below basic literacy skills, we will reduce the reading level of the nutrition manual and use visual recipes, to ensure returning citizens with poor reading skills will be able to successfully use it [12]. Second, we will supplement the manual with information relevant to individuals with low socioeconomic status living in transitional housing settings. We will incorporate information from the Homeless Nutrition Education Toolkit developed by the Sacramento Hunger Coalition and the Snap-Ed toolkit for Homeless/Pantry Clients [19, 20]. These modifications will allow us to deliver a curriculum that emphasizes eating healthy from food pantries, grocery shopping using public food assistance (i.e., EBT or food stamps), and preparing healthy meals without a kitchen or refrigerator. Community-based reentry program staff will create a comprehensive and detailed manual that outlines the objectives, content, activities, and resources for implementing the curriculum. They will also incorporate nutrition information into their smartphone app (available for Android and iPhone) and website.

Interactive activities will be important components of our program. From our previous experience in running a nutrition program, we learned that (1) participants learn best by preparing healthy meals in the classroom setting, and (2) competitive challenges encourage class attendance and positive behavior change. The program will, therefore, include cooking classes during which participants can practice preparing simple meals that do not require a kitchen. Additionally, the program will offer prizes (e.g., gift cards to the local farmer’s market, water bottles, sports towels, physical activity trackers) for participants who win weekly challenges (e.g., water challenge, fruit/vegetable challenge).

### Physical activity and nutrition program

Eligible participants are formerly incarcerated adults aged 18 + who are current or former participants of a local community reentry program. This program provides wrap-around services for formerly incarcerated adults, including reentry care packages (e.g., toiletries, underwear, bed linen), transitional housing, job training, and life skills training (e.g., financial literacy, parenting, coping skills). Formerly incarcerated adults aged 18 + who are current or former participants in the community-based reentry program will be eligible to participate in the physical activity and nutrition program. We will enroll up to 15 formerly incarcerated adults in the program. This is a non-randomized feasibility study; thus we did not perform formal sample size calculations. Rather, we will aim for a sample size of 15 participants as a pragmatic decision based on availability of resources. Participants will not be restricted from participating in any other community programs or interventions while participating in this program.

Participants will participate in weekly nutrition classes that will take place at the community-based reentry program’s office. Classes will be run by a local nutritionist using an apprenticeship model, where one to two participants assist and provide support during the class. We expect that this apprenticeship model will boost local program capacity to support nutrition education for returning citizens in the future. Of the 16 1-h classes in the program, eight classes will focus on general nutrition education, four classes will be food preparation classes, and four classes will be special topics geared toward the unique needs of returning citizens (e.g., grocery shopping using public food assistance, eating well from food pantries, preparing meals without a kitchen). We will offer options for day and evening classes to accommodate the schedules of program participants. Program participants will be given city bus passes to ensure their ability to attend classes.

Physical activity classes will take place weekly over the 16-week program period. The four individuals trained as group fitness instructors will lead two classes a week that include a warm-up, moderate intensity aerobics, resistance training with cuff weights, flexibility training, and a cool down. Additionally, participants will be asked to engage in at least 30 min of physical activity twice a week in addition to the weekly physical activity class. We also will provide participants with six-month memberships to a local fitness center.

We will discontinue or modify fitness program components if participants develop medical conditions during the study that limit their ability to exercise safely. For example, participants will have the option to engage in seated and/or low impact versions of all exercises. Unexpected adverse events (AEs) that occur during the intervention period will be reported to the study team. Serious adverse events will be reported to the Principal Investigator and the IRB immediately in order to determine whether participants should withdraw from the study.

### Supportive training component

Social support is an important predictor of chronic-disease related lifestyle change [21, 22]. We will utilize two approaches for increasing social support for program participants. Each participant will be able to invite a “buddy” to participate in the program. These individuals will be able to participate in nutrition and physical activity classes along with the participants. Further, to attend to the unique needs of returning citizens, two formerly incarcerated individuals will be appointed to serve as peer mentors to program participants. These individuals will be able to provide general support related to community reentry for recently incarcerated participants.

### Program evaluation

Research staff will lead program evaluation efforts for the project. Primary outcomes include reach, preliminary effectiveness, adoption, implementation, and maintenance (Table 2).

**Table 2:** Summary of program evaluation components and measurement outcomes

Components	Measurement
Reach	Fitness instructor training program The number of potential fitness instructors who complete certification The number of fitness instructors who successfully teach in the program Physical activity and nutrition program Number of program participants reached Representativeness of participants reached Mean number of sessions attended by participants The number of participants who complete the program
Effectiveness (preliminary)	Fitness instructor training program Trainee scores on fitness training certification exams Physical activity and nutrition program Participant scores on psychosocial, behavioral, and physical health outcomes
Adoption	Fitness Instructor Training Program Barriers and facilitators to adoption of the training program Physical activity and nutrition program Barriers and facilitators to adoption of the physical activity and nutrition program
Implementation	Fitness instructor training program Training satisfaction surveys and focus groups Physical activity and nutrition program Fidelity checklists to evaluate if physical activity and nutrition program delivered as intended Program satisfaction surveys and focus groups
Maintenance	Fitness instructor training program Fitness trainee continued involvement in group fitness instruction at six months Physical activity and nutrition program Psychosocial, behavioral, and physical health outcome maintenance at six months

#### Reach

We will evaluate the reach of the fitness instructor training program and the physical activity and nutrition program. Reach-related outcomes for the fitness instructor training program include the number of potential fitness instructors who complete fitness instructor certification and the number of fitness instructors who successfully teach in the program. Reach-related outcomes for the physical activity and nutrition program will include the number of community-based reentry program participants reached, the representativeness of program participants reached, the mean number of physical activity and nutrition program sessions attended by participants, and the number of participants who complete the program.

#### Effectiveness

We will collect survey and physical measurement data at baseline, 16 weeks (end of program) and at a six-month follow-up (Table 3). Participants will be paid $20 for participation in each assessment. Assessments will take place at the community-based reentry program office. We will calculate means and distributions of all study variables at baseline, 16 weeks, and 6 months.

**Table 3:** Summary of survey data to be collected

Psychosocial variables	
Anxiety	Generalized Anxiety Disorder Scale [23]
Depressive symptoms	Patient Health Questionnaire-9 [21]
Self-efficacy for nutrition	Diet Self-Efficacy Scale [22]
Self-efficacy physical activity	Self-Efficacy for Physical Activity Scale [24]
Social support for physical activity	Social Support for Physical Activity Scale [25]
Social support for nutrition	Social Support for Nutrition Scale [25]
Health behaviors	
Smoking	Past and current smoking, cigarettes per day
Sleep habits/quality	PROMIS Sleep Short Form [26]
Fruit/vegetable intake	Diet History Questionnaire [25]
Physical activity	International Physical Activity Questionnaire [27, 28]
Self-reported physical health status	
Self-reported chronic pain	Brief Pain Inventory [29]
Self-rated health/health related quality of life	Promis Global Health Scale [30]
Medication usage	Use of medications for chronic conditions
Self-reported chronic health conditions	Hypertension, heart disease, arthritis, cancer, diabetes, lung disease, stroke

After informed consent, participants will be asked to complete a 20-min survey that collects psychosocial data, information on health behaviors (e.g., physical activity, dietary patterns, tobacco/alcohol use, sleep) and health status. As needed, surveys will be read aloud to accommodate all reading levels. Physical measurements will be collected by research project staff. Program participants will provide their contact information to study staff at baseline. Those who discontinue the program will be contacted by phone and asked to complete outcome assessments either by phone (e.g., surveys) or in-person (e.g., physical assessments).

*Demographic measures* will include age, gender, marital status, parental status, education, and employment status. *Psychosocial measures* will include measures of depressive symptoms, anxiety, self-efficacy for physical activity and nutrition, and social support for physical activity and nutrition.

*Health behaviors* will be assessed, including tobacco use, alcohol use (e.g., number of drinks per week), and sleep behaviors (e.g., sleep quality, hours of sleep per night). *Health status measures* will include self-reported health conditions (e.g., diabetes, hypertension, arthritis, high cholesterol, heart disease), self-reported chronic pain, overall self-rated health, and health-related quality of life.

*Physical activity measures* will include minutes of vigorous/moderate/light exercise per week, and frequency of physical activity. *Diet and nutrition measures* will include evaluation of fruit and vegetable intake, water consumption, food security (i.e., availability of healthy foods), and fast food consumption.

##### Physical measurements

Systolic and diastolic blood pressure, weight, height, and waist circumference will be collected by research staff. Weight and height data will be collected from participants without shoes, and participants will be asked to wear no more than one lightweight layer of clothing during weight measures. After resting quietly in a seated position for 5 min, three consecutive heart rate and blood pressure readings will be obtained using an automated oscillometric blood pressure measuring device. If a blood pressure measurement is interrupted or incomplete, a fourth attempt may be made. Weight will be ascertained using digital scales. Height and waist circumference will be measured using a fiberglass measuring tape.

#### Adoption

We will use qualitative methods to understand adoption. We will participate in weekly meetings with program staff before and after initiation of the fitness training program and the physical activity and nutrition program. We will use information from these meetings to gather information on adotpion barriers and facilitators. Meetings will be recorded, and barriers and facilitators will be documented in meeting minutes.

#### Implementation

We will evaluate implementation of both the fitness instructor training program and the physical activity and nutrition program.

##### Implementation of fitness training program

After completing informed consent, trainees will complete surveys that assess their (1) overall satisfaction with instructor training, and (2) perceived ability to handle the tasks, obligations, and challenges associated with group fitness instruction (self-efficacy). Self-efficacy assessments will, in particular, assess their confidence in their ability to perform tasks related to the five components of our training program (e.g., ability to design and teach fitness classes, perform chest compressions, perform mouth-to-mouth rescue breathing, use a defibrillator, perform basic first aid, and provide instruction in moderate aerobic exercise/flexibility/resistance training).

We will calculate means and standard deviations for all domains of the quantitative assessments. Quantitative data will then inform the direction of the trainee focus group. In particular, we will use the scores on the quantitative assessments to identify the areas where trainees felt most challenged during the training process and solicit recommendations for handling those challenges going forward.

We will conduct three, 1-h focus groups with the four trainees who participated in fitness instructor training program. We will develop a structured interview guide that allows fitness trainees to expound on their satisfaction with the training process. We will also query them regarding any difficulties they experienced in getting trained, and any changes to the training process that would be beneficial. We will discuss potential approaches for increasing self-efficacy across the various components of the training program.

We will conduct two focus groups with the fitness coach, the project research assistant, and community-based reentry program staff. We will use these focus groups to get trainer/staff perspectives on (1) trainee readiness to serve as fitness trainers and (2) the process of working with trainees during the training process. We also will share with this group the key findings gleaned from the trainee focus group, including their recommendations for improving the training process and increasing trainee self-efficacy.

All qualitative interviews will be recorded, transcribed verbatim, and de-identified prior to analysis. We will use the domains covered by our survey measures as a deductive coding framework and then identify inductive codes as they arise. Data will be coded using Dedoose, a web-based qualitative data analysis program [31]. Two coders will independently code the interview transcripts. Coding results will then be compared for consistency. When interrater agreement is not achieved, the two coders will meet to discuss findings and to reach final agreement.

We will use our quantitative and qualitative data to identify areas where trainees may need additional support to maximize their skills as trainers. As needed, the fitness consultant will work with the trainees to provide supplemental “booster” training to sharpen trainees’ skills. Additionally, we will use these data to develop recommendations for addressing any challenges that arise from training returning citizens as fitness instructors. This information will be useful for future replication efforts.

##### Implementation of physical activity and nutrition program

To monitor implementation, we will create fidelity checklists to evaluate the extent to which the physical activity and nutrition programming is delivered as attended [24, 32]. The fidelity checklists will measure instructor adherence to session content, quality, participant engagement, and responsiveness to feedback. The project research assistant will complete these checklists during each physical activity and nutrition class. In addition to tracking activities, the research assistant also will document any problems that occurred during the class and how those problems were resolved. The research team will attend weekly programmatic meetings to document any modifications or adaptations recommended by the program staff and to document the impact of those modifications/adaptations on program delivery. We also will measure participant *satisfaction* with and *usage* of various program components. This includes satisfaction/usage of: support mentors, the nutrition smartphone app, fitness classes, the nutrition curriculum, pantry usage and grocery shopping tips, and meal preparation strategies learned in cooking workshops. This will be done in two ways: first, participants will complete brief quantitative measures that capture data on usage and satisfaction. Second, research staff will conduct short focus groups with program participants at weeks 4, 8, and 16 of the intervention to discuss program components and ways to improve the intervention.

### Maintenance

To evaluate maintenance with respect to the fitness instructor training program, we will evaluate the extent to which trained fitness instructors continue to be engaged as group fitness instructors at the 6-month data collection point. We will use survey and physical health assessment data collected at six months to evaluate maintenance with respect to the physical activity and nutrition program.

### Data management and monitoring

Data management will be conducted by an experienced team, directed by the Principal Investigator. Each study participant will be issued an ID number that will be used for data collection. Quantitative survey data will be collected electronically and will only be identified with the study ID number of the participant. Codes linking participant names with study ID numbers will be kept confidential in secured electronic files on password-protected servers only accessible to the research team. We will collect quantitative data via direct electronic data capture using Qualtrics, a secure, HIPAA compliant, web-based application. In circumstances where direct electronic data capture is not possible, we will use paper-based study forms. Completed study forms will be stored in a locked drawer in the PI’s locked office. We will use standard data checking procedures to check all forms for missing data. Standard data checking procedures will include checking forms for missing data, double entry with discrepancy resolution, daily back-up copies of computer files, and examination of key variables for skewness, variability, missing data, and outliers.

Qualitative interviews and focus groups will be audio-recorded using a password-protected encrypted digital voice recorder. All recordings will be stored on a password-protected computer.

After interviews are complete, recordings will be de-identified, encrypted and sent to a third-party transcription service.

### Protocol changes

All protocol changes will be submitted to the IRB as modifications prior to implementation. Changes will also be communicated during dissemination to both academic and lay audiences (see “Dissemination plans” section).

### Dissemination plans

We will disseminate study findings to academic and lay audiences. We will create one-page fact sheets that summarize major findings. These fact sheets will be given to study participants and posted on the reentry program’s website. Additionally, we will invite participants to a culminating event at the end of the study where we will share study findings. We will disseminate study findings at academic conferences and via academic publications. The sponsor has imposed no publication restrictions.

### Progression criteria and future study

Progression criteria for this study are related to recruitment, acceptability, and completeness of outcome data. The next step from this study will be a pilot randomized controlled trial (RCT) comparing the physical activity and nutrition program to a treatment-as-usual control. We will continue to a pilot RCT if (1) we recruit and consent at least 15 participants in the feasibility study, (2) 70% of enrolled participants report satisfaction with the program via survey assessments, and (3) we obtain complete outcome data from at least 70% of enrolled participants at 16 week follow-up.

## Discussion

This project is innovative in several ways. It will be one of only a few studies designed to specifically address preventive and non-pharmaceutical physical health improvement among returning citizens. Few programs and research studies focus on physical health among returning citizens. Most interventions conducted in this population focus on mental health, substance abuse, and job readiness [4]. When physical health interventions are conducted, they largely address clinical care (e.g., pharmaceutical treatment of ongoing medical problems) [5]. Our project addresses nutrition and physical activity—two known predictors of physical health outcomes and chronic disease onset.

Our project is designed to increase the capacity of returning citizens (and the organizations serving them) to promote physical health. One of the project’s core components is increasing individual and organizational capacity to support physical activity and nutrition programming among returning citizens. By training returning citizens to provide group fitness instruction and working with reentry programs to develop and implement an appropriate nutrition program for this population, we are investing our efforts and resources in activities that can be sustained after project funding is complete.

This project promotes health equity by addressing the unique nutritional needs of returning citizens. Traditional community-based nutrition programs fail to consider how housing insecurity and poverty impact an individual’s ability to engage in healthy eating. By developing and implementing a nutrition curriculum that intentionally addresses these issues, we remove some of the obstacles to heathy eating, thus increasing healthy equity in this population.

## Conclusions

Healthy eating and physical activity depend on numerous social determinants of health, including food access, adequate housing, community/social support, health literacy, and income [33]. Returning citizens typically have gaps in all these areas. We will use this feasibility study to test our ability to address these social determinants in our physical activity and nutrition programming designed specifically for returning citizens. Additionally, by training formerly incarcerated adults to serve as fitness trainers, our work will build long-term capacity for health and wellness within a community-based reentry program and provide fitness trainers with a skill that can then be leveraged for future employment and income opportunities. We will use the current study to inform a future pilot RCT that will test the acceptability of our randomization procedures, estimate effect sizes, and calculate statistical power in preparation for a fully powered RCT.

## Data Availability

Data sharing is not applicable to this article as no datasets were generated or analyzed during the current study.
